# Effect of late sodium current inhibition on MRI measured diastolic dysfunction and myocardial perfusion reserve in aortic stenosis: a pilot study

**DOI:** 10.1186/1532-429X-17-S1-P320

**Published:** 2015-02-03

**Authors:** Anvesha Singh, Jamal N Khan, Christopher D Steadman, Michael Jerosch-Herold, Sheraz A Nazir, Prathap Kanagala, Gerry P McCann

**Affiliations:** 1 Cardiovascular Sciences, University of Leicester and the NIHR Leicester Cardiovascular Biomedical Research Unit, Leicester, UK; 2 Cardiology, Poole Hospital NHS Foundation Trust, Poole, UK; 3 Brigham and Woman's Hospital and Harvard Medical School, Boston, MA, USA

## Background

Aortic Stenosis (AS) is characterized by pressure overload hypertrophy (LVH) with associated diastolic dysfunction. The presence of LVH, diastolic dysfunction and microvascular dysfunction appear to be important determinants of exercise capacity in AS. There are no medical therapies in AS of proven value. Ranolazine is licenced for the treatment of angina and inhibits late sodium channel activation. Ranolazine has been shown to improve diastolic dysfunction in isolated myocytes.

## Methods

In this prospective, open label study with blinded endpoint analysis, patients with asymptomatic moderate/severe AS and diastolic dysfunction or LVH were recruited. Patients, underwent trans-thoracic echocardiography, exercise testing and adenosine stress cardiac magnetic resonance imaging at baseline, 6 weeks after commencing Ranolazine (maximum dose 750mg BD) and again at 10 weeks (4 weeks after discontinuation). Tagged images were acquired at three short-axis slices on a 3T platform (Siemens Skyra). Myocardial perfusion reserve was calculated from stress and resting blood flow. The primary hypothesis was that Ranolazine would improve peak early diastolic strain rate (PEDSR) on tagged MRI.

## Results

Fifteen patients (PPG 48.8±12.4 mmHg, MPG 27.1±7.5 mmHg, AVA 1.26±0.31 cm^2^, LV mass index 66.72±15.35 g/m^2^) completed the week-6 visit and 13 completed the final visit. Results are shown in table 1. There was a trend for the global PEDSR to increase from the baseline to week-6 (0.79 ± 0.15 to 0.86 ± 0.18, p=0.198). For those who completed the final visit, PEDSR increased from baseline to week-6 and then returned close to the baseline value at week-10 (table). There was no significant change in MPR or echocardiographic measures of diastolic dysfunction. The total exercise duration increased from 10.47±3.68 minutes to 11.60±3.25 minutes (n=15, p=0.06), with a trend for the maximal HR and SBP to be lower at week-6, resulting in a reduction in exercise LV rate-pressure product (LVRPP), suggesting improved myocardial efficiency. On splitting the patients into low and high-MPR subgroups based on the median MPR, the trend for the improvement in PEDSR (0.88 ± 0.80, 1.03 ± 0 .30, 0.86 ± 0.15) and reduction in exercise LVRPP was maintained in the low-MPR subgroup only.

**Table 1:** Change in primary and secondary endpoints with Ranolazine

Parameter	Baseline	Week-6	Week-10
MRI parameters			

PEDSR (1/s)	0.82 ± 0.130	0.87 ± 0.193	0.81 ± 0.211

MPR	2.69 ± 0.726	2.45 ± 0.559	2.52 ± 0.579

Exercise Parameters			

Resting HR (bpm)	74.1 ± 12.0	73.5 ± 13.2	72.0 ± 11.7

Resting SBP (mmHg)	155.9 ± 24.0	148.5 ± 17.9	146.2 ±25.0

Exercise duration (min)	10.88 ± 3.94	11.85 ± 3.39	11.99 ± 3.59

Max HR (bpm)	142.2 ± 11.0	136.5 ± 13.7	140.6 ± 12.7

Max SBP (mmHg)	183.9 ± 20.6	174.5 ± 24.2	179.3 ± 15.1

Resting LVRPP (mmHg.bpm)	14639.7 ± 3228.3	14735.9 ± 3773.6	14512.6 ± 3131.8

Exercise LVRPP (mmHg.bpm)	36342.3 ± 5351.4	34285.9 ± 6902.7	35604.6 ± 4787.5

Echocardiographic parameters			

E/A	0.742 ± 0.143	0.765 ± 0.171	0.775 ± 0.159

Septal E/e'	12.57 ± 3.81	13.50 ± 2.95	13.84 ± 3.85

Lateral E/e'	10.93 ± 3.48	10.90 ± 3.37	11.03 ± 2.75

PEDSR: peak early diastolic strain rate, MPR: myocardial perfusion reserve, HR: heart rate, SBP: systolic blood pressure, LVRPP: left ventricular rate pressure product. All p>0.05.

## Conclusions

This pilot hypothesis-generating study has shown some signals towards Ranolazine improving diastolic function and exercise myocardial efficiency in patients with AS, particularly in those with a lower MPR. The current results would support a larger study in patients with diastolic dysfunction.

## Funding

This study was funded and sponsored by Menarini International Operation, Luxembourg with support from the NIHR Leicester Cardiovascular Biomedical Research Unit (CVBRU). GPM is funded by an NIHR research fellowship and AS by the NIHR Leicester CVBRU.

**Figure 1:**
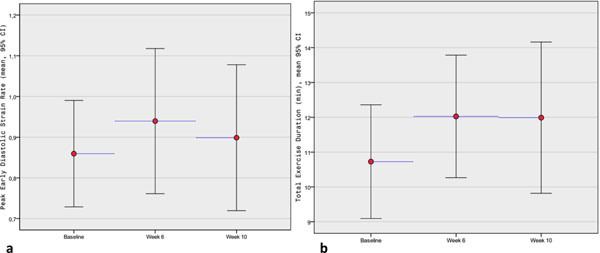
**a.)Trend in PEDSR with study visit for the Basal slice, b.) Total exercise duration for all patients exercised at each visit** (p=0.07 for baseline *vs* week-6, p=0.73 for week-6 *vs* week-10)

